# Editorial: Stomatal Biology and Beyond

**DOI:** 10.3389/fpls.2022.848811

**Published:** 2022-02-11

**Authors:** Wenxiu Ye, Juan Dong, Toshinori Kinoshita

**Affiliations:** ^1^Shanghai Collaborative Innovation Center of Agri-Seeds, Center for Viticulture and Enology, School of Agriculture and Biology, Shanghai Jiao Tong University, Shanghai, China; ^2^Shanghai Urban Forest Ecosystem Research Station, National Forestry and Grassland Administration, Shanghai, China; ^3^Waksman Institute of Microbiology, Rutgers, The State University of New Jersey, Piscataway, NJ, United States; ^4^Department of Plant Biology, Rutgers, The State University of New Jersey, New Brunswick, NJ, United States; ^5^Institute of Transformative Bio-Molecule, Nagoya University, Nagoya, Japan; ^6^Graduate School of Science, Nagoya University, Nagoya, Japan

**Keywords:** stomata, guard cell, biotechnology, development, signaling

Stomata, each surrounded by a pair of guard cells, are microscopic pores in the shoot epidermis of plants, which serve as a low-resistance pathway for the diffusional movement of gas and water vapor between a plant and the environment. On the other hand, as natural openings in the leaf, stomata are exploited as a convenient entry route by a wide range of pathogens. In adaption to the ever-changing environment, guard cells have acquired sensing mechanisms to a variety of internal and external stimuli resulting in the change of turgor pressure in guard cells. As a result, stomata open and close, by which the plant actively regulates gas exchange with the environment including CO_2_ uptake and over 90% water transpiration, and wards off pathogens. Therefore, stomata have a central impact on regulating plant photosynthesis, water status, and stress resistance at the plant physiology level, and on crop production as well as the global carbon and water cycle at the social-ecological level. Stomatal biology has been consistently one of the major research fields of plant science and develops rapidly as evidenced by several research collections on this Research Topic (Blatt et al., 2017; Raghavendra and Murata, 2017; McAdam et al., 2021) as well as the 15 articles in this Research Topic. These Research Topics have provided great insights into the fundamental mechanisms underlying stomatal movement, development, and patterning at both the molecular and systemic levels, making guard cell one of the best-characterized model systems for plant cell and developmental biology. There is also increasing interest in the translation of the knowledge to crop production. The present Frontiers Research Topic covers recent research and review papers on the Research Topics of stomatal development, stomatal dynamics in response to environmental cues, and stomatal manipulation technologies.

Stomate is a fascinating model for developmental biology, not only for its highly specialized division and differentiation processes, also for the formation of isolated guard cells without plasmodesmata. The asymmetric divisional behavior and seemingly random yet one-cell-spaced distribution pattern provide an ideal system for studying cell polarity and cell-division orientation (Pillitteri and Dong, 2013). In this Research Topic, Han et al. overview the recent advances in understanding the molecular basis of cell fate and dynamics of stomatal lineage cells at the cell state- or single-cell level and the regulation of stomatal development by environmental cues. Xiao et al. performed a comprehensive functional analysis of *Arabidopsis* guard cell-enriched GDSLs Lipases (GGLs) and determined several GGLs are involved in regulating stomatal density and morphology. Stomata in dicot and monocots are different in morphology with kidney shape for dicots and dumbbell shape for monocots. In addition, the monocot stomatal complex consists of two subsidiary cells surrounding two guard cells. Therefore, research on monocots is necessary to fully understand stomatal development. Based on a forward genetic screening, Yu et al. find a protein with unknown function, Rice Stomata Development Defect 1, is important for stomatal development in rice and appears to regulate proper expression of the protease gene, *Stomatal Density and Distribution 1*. Serna provides a perspective on the functions of a master regulator, the MUTE transcription factor, in the last step of stomatal development in monocots.

Stomatal movement responding to environmental stimuli including light, CO_2_, and dehydration, is a major research area of stomatal biology because of their important role in photosynthesis and water use efficiency. In this Research Topic, Liu et al. find that a cyclophilin, ROC3, positively regulates the stress hormone abscisic acid-induced stomatal closure and dehydration response by regulating reactive oxygen species homeostasis and transcription of stress-response genes. Several GGLs identified by Xiao et al. are also involved in stomatal dynamics and plant water relations. In terms of light signaling in guard cells, Ye et al. identify several new genes including a vacuolar channel, Aluminum-Activated Malate Transporter 6, required for in blue light-induced stomatal opening. A pharmacological study by Wang et al. suggests that a group of proteinases are required for light signaling in guard cells. The stomatal responses are quite different in crassulacean acid metabolism (CAM) and C3 plants. A typical difference is that CAM stomata open in the dark and close in response to light, while C3 stomata operate in an opposite way. In this Research Topic, Santos et al. investigated the stomatal response to light and CO_2_ in C3 and CAM plants, using *Vicia faba* and *Kalanchoë fedtschenkoi* as model plants. This elegant study provides evidence that both signals from guard cells and mesophyll contribute to the differences observed in CAM and C3 stomatal responses. Research on stomatal immune responses against pathogens has been very active these years with a focus on the short-term defenses (Thor et al., 2020; Ye et al., 2020). Here, Gahir et al. raise an interesting point on the long-term effect of stomatal closure in terms of defense against pathogen and discuss possible research directions.

One important goal of research on stomatal biology is, by developing new strategies to manipulate stomatal development, activity and/or physiology, to improve crop resilience and water use efficiency. To this end, there is also a trend shifting from using model plants to crops for research. In this Research Topic, Hayashi et al. constructed a gene expression database of *V. faba* and found ABA-dependent phosphorylation of the *V. faba* orthologs of important genes known to be involved in the stomatal movement including a basic helix–loop–helix transcription factor *VfAKS1*, an ABA importer in guard cells *VfABCG40*, and a clathrin heavy chain *VfCHC1*. These results set the basis for future research on stomatal biology in *V. faba*. Pitaloka et al. reveal the functions of rice stomatal mega-papillae in stomatal dynamics, defense against pathogens, highlighting its potential as an important trait for rice breeding. Stomatal phenotyping is critical for field study, which is greatly facilitated by the emerging machine-learning-based imaging analysis. Zhu et al. build up a method based on machine learning to automatically assess the stomatal index in wheat. Moreover, Toda et al. report the establishment of a readily available image-analysis platform to accelerate stomatal phenotyping in the field. Regarding manipulation approaches of stomatal traits, agrochemicals have been proved very effective (Kinoshita et al., 2021). The protease inhibitors (PIs) identified by Wang et al. could be the lead compounds for manipulating stomatal movement. A more efficient and environmental-friendly approach would be genetic technology. The guard cell-specific promoters identified by Ye et al. are a plus to the toolkits for designing guard cell-targeting genetic strategies. A beautiful example to show the contribution of genetic manipulation of stomata to crop production is the establishment of the “PUMP” technique short for the promotion and upregulation of plasma membrane proton-ATPase (Zhang et al., 2021). By increasing the expression level of plasma membrane H^+^-ATPases in guard cells, this strategy has been proved very efficient for enhancing photosynthesis and thus plant growth. In this Research Topic, Ren et al. review the progress and synthesize the principle of this technique. Toh et al. report the technique is also efficient in enhancing photosynthesis and growth of perennial woody plants using poplar as a model.

## Concluding Remarks

Stomate is an evolution success for plants to colonize land some 450 million years ago. Because of its importance to plant physiology, there is continuing scientific effort to understand its evolution path, mechanism of its biogenesis and function, and potential for translation. The current Research Topic accommodates new findings on most of these areas and is dedicated to stimulating future research on stomatal biology. More research efforts are needed on new research directions, such as guard cell metabolism, cell wall mechanics, memory mechanism, as well as on many economically important plants. Particularly, we want to highlight two research areas under development, stomatal traits influenced by biotic stimuli and pollutants from human activity, because of their importance in crop protection and climate control. Regarding stomatal biotechnology, we have witnessed striking progress as a result from combining approaches of genetics, optics, and chemistry (Papanatsiou et al., 2019; Kinoshita et al., 2021). These research findings underline the importance of incorporating knowledge from multi-disciplines to realize innovation with broad impact. We believe stomatal biotechnology is a powerful strategy to achieve sustainable development goals including crop production improving and climate control. Bearing this in mind, this Research Topic is delivered as a call for more participation of scientists from different fields.

## Author Contributions

WY wrote the first draft. All authors contributed to the article and approved the submitted version.

## Funding

This work was supported by the National Natural Science Foundation of China (31901984 to WY), the National Institute of Health (GM131827 to JD), the National Science Foundation (1851907, 1952823, and 2049642 to JD), and Grants-in-Aid for Scientific Research from the Ministry of Education, Culture, Sports, Science, and Technology, Japan (20H05687 and 20H05910 to TK).

## Conflict of Interest

The authors declare that the research was conducted in the absence of any commercial or financial relationships that could be construed as a potential conflict of interest.

## Publisher's Note

All claims expressed in this article are solely those of the authors and do not necessarily represent those of their affiliated organizations, or those of the publisher, the editors and the reviewers. Any product that may be evaluated in this article, or claim that may be made by its manufacturer, is not guaranteed or endorsed by the publisher.
